# Supplementary material for: Prevalence and determinants of medicinal plants utilization during labour among women of reproductive age in Butiama, Tanzania: A community-based cross-sectional study
Source: PLoS One. 2025 Oct 31;20(10):e0334453. doi: 10.1371/journal.pone.0334453 (PMC12578257; doi:10.1371/journal.pone.0334453)
Supplement: S1 File — (DOCX) [file pone.0334453.s002.docx]

**TITLE**: **Determinants of Herbal Medicine Utilization during Labour among Women of Reproductive Age in Mara, Tanzania**

**SOCIO-DEMOGRAPHIC INFORMATION**

| **S/N** | **Question** | **Option/response** | **Remark** |
| --- | --- | --- | --- |
|  | What is your age (years) | … |  |
|  | What is your marital status now? | 1. Single  2. Married/cohabiting  3. Widowed/separation/Divorced |  |
|  | What is your education level? | 1. None/primary incomplete. 2. Primary complete. 3. Secondary and above. |  |
|  | What is your monthly income? (Tzs.) | 1. < 100,000  2. 100,000-500,000  3. ≥ 500,000 |  |
|  | What is your occupation? | 1. Peasant/housewife  2. Self-employed  3. Formerly employed  4. Employed in the formal sectors |  |
|  | What is the education level of your partner? | 1. None/primary incomplete. 2. Primary complete. 3. Secondary and above. |  |
|  | How far away is the nearest healthcare facility in kilometres? | 1. > 5  2. ≤ 5 |  |
|  | How many antenatal care (ANC) visits did you attend during your most recent pregnancy? | 1 < 4  2. ≥ 4 |  |
|  | How many children have you given birth to (parity)? | 1. one  2. 2-5  3. >5 |  |
|  | Have you ever experienced the loss of a child through death? | 0. Yes  1. No |  |
|  | Where did you give birth during your last pregnancy? | 1. Home  2. Institution |  |
|  | Do you/your family own the following items; ((Select all that apply))  1. Radio  2. TV  3. Mobile phone  4. Computer  5. Refrigerator  6. Bicycle  7. Animal Drawn Cart  8. Motorcycle/Scouter  9. Car  10. None of the above | 0 = Lowest economic status (own none of the possessions or only radio),  1 = Second (radio, phone, bicycle),  2= Middle (radio, TV, mobile phone, bicycle)  3 = Fourth (radio, TV, mobile phone, refrigerator, bicycle, animal-drawn cart, motorcycle/scouter)  4 = Highest economic status (owns all in the list with a Car) |  |

**Herbal medicines used during labour among women of reproductive age.**

| **S/N** | **Question** | **Option/response** | **Remark** |
| --- | --- | --- | --- |
|  | Which of the following herbal medicines have you used during labour in any of your pregnancies? (Select all that apply) | 1. Pumpkin roots (Cucurbita pepo)  2. African spider plant (Cleome gynandra)  3. Ginger (Zingiber officinale Roscoe)  4. Concentrated green or black tea (Camellia sinensis)  5. Never used herbal medicine |  |
|  | Herbal medicines were used during labour in any of your pregnancies.  (If mother did not used herb medicine during labour go to qn. 19) | 0. yes  1. No |  |
|  | What are the reasons for your intake of herbs during labour? | 1. To enhance labour  2. To keep the foetus healthy  3. To relieve pain  4. Other (prevent constipation & vomiting) |  |
|  | Did you experience any adverse effects or complications as a result of using herbal medicine during labour? | 0. Yes  1. No |  |
|  | How did you first learn about the use of herbal medicine during labour? | 1. Family members or relatives  2. Traditional birth attendants  3. Friends or neighbors  4. Community health workers  5. Other (please specify): _______________ |  |
|  | What factors, in your opinion, influenced your decision to use herbal medicine during labour? (Select one or more) | 1 Cultural beliefs and traditions  2. Lack of access to modern healthcare facilities  3. Perceived effectiveness of herbal medicine  4. Perceived safety of herbal medicine  5. Advice from family or community members  6. Advice from healthcare providers  7. Cost of modern medicines  8. Other (please specify): _______________ |  |
|  | How easily accessible and available are herbal remedies in your local community? | 1. Very Inaccessible  2. Inaccessible  3. Neutral  4. Accessible  5. Very Accessible |  |

**Likert scale of perception of herbal medicine use among women of reproductive age**

| **S/N** | **Questions** | strongly disagree | Agree | Neutral | Disagree | strongly Disagree |
| --- | --- | --- | --- | --- | --- | --- |
|  | Do you consider herbal medicines to be safer than modern medical interventions during labour? |  |  |  |  |  |
|  | Do you believe that herbal medicines are more effective in managing labour pain compared to modern medical interventions? |  |  |  |  |  |
|  | Do you trust the safety of modern medical interventions during labour? |  |  |  |  |  |
|  | To you, are modern medical interventions the preferred choice for ensuring a safe childbirth experience? |  |  |  |  |  |
|  | Would you recommend herbal medicines to other women for managing labour pain? |  |  |  |  |  |
|  | Do your cultural beliefs influence your perception of the effectiveness of herbal medicines during labour? |  |  |  |  |  |
|  | Do you believe that healthcare providers have sufficient knowledge about the safety of herbal medicines during labour? |  |  |  |  |  |
|  | Do you feel well-informed about the potential risks associated with herbal medicine use during labour? |  |  |  |  |  |
