# Supplementary material for: Prevalence and determinants of medicinal plants utilization during labour among women of reproductive age in Butiama, Tanzania: A community-based cross-sectional study
Source: PLoS One. 2025 Oct 31;20(10):e0334453. doi: 10.1371/journal.pone.0334453 (PMC12578257; doi:10.1371/journal.pone.0334453)
Supplement: S1 Checklist — (DOCX) [file pone.0334453.s003.docx]

Inclusivity in global research

PLOS’ policy on inclusivity in global research aims to improve transparency in the reporting of research performed outside of researchers’ own country or community and ensures that PLOS publications reporting global research adhere to high standards for research ethics and authorship. Authors of relevant research articles may be asked to complete the questionnaire below, which outlines ethical, cultural, and scientific considerations specific to inclusivity in global research. This questionnaire may be requested when researchers have travelled to a different country to conduct research, if research uses samples collected in another country, research with Indigenous populations or their lands, or if research is on cultural artefacts. Researchers travelling to another country solely to use laboratory equipment will not normally be required to complete the questionnaire. However, the questionnaire can be requested at the journal’s discretion for any submission – if you have been requested to complete this questionnaire by the PLOS journal you submitted to, please do so.

Please complete the questionnaire below and include this as a Supporting Information file with your manuscript. Note that if your paper is accepted for publication, this checklist will be published with your article in the supporting information files. Please ensure that you reference the checklist in the main body of your manuscript. We suggest adding a subsection ‘Inclusivity in global research’ to your Methods section and adding the following sentence: “Additional information regarding the ethical, cultural, and scientific considerations specific to inclusivity in global research is included in the Supporting Information (SX Checklist)”

The questions have been designed to be applicable to a wide range of study types, and there are subsections for both human subjects research and non-human subjects research. If any of the questions are not relevant to your research please mark them as “N/A” as appropriate.

**Ethical considerations, permits and authorship**

*This section is applicable to all research types.*

Provide details as to who granted permissions and/or consent for the study to take place in the Methods section of your manuscript. This should include the names of **all** ethics boards, governmental organizations, community leaders or other bodies that provided approval for the study. If individuals provided approval refer to these people by their role or title but do not list their name(s).

Reported on page number: 09

If there were any deviations from the study protocol after approval was obtained please provide details of these changes in the Methods section of your manuscript.
Did this study involve local collaborators that are residents of the country where the research was conducted or members of the community studied? If you do not have any authors from said communities, please provide an explanation for this below.

Reported on page number: N/A

**Involvement of Local Collaborators:**

Yes, this study involved local collaborators who are residents of the country where the research was conducted. The Principal Investigator (PI) is a Tanzanian national and was responsible for coordinating field activities, community engagement, and data collection. In addition, two other main co-authors are also based in Tanzania and contributed to various aspects of the study. While two co-authors are based in China, their contributions focused on study design, data analysis, and manuscript preparation. The strong involvement of local researchers ensured that the study remained contextually relevant, ethically sound, and culturally appropriate.

Everyone listed as an author should meet PLOS’ criteria for authorship and all individuals who meet these criteria should be included in the author byline, rather than the acknowledgements. For further information please see the journal’s Authorship Policy.

**Human subjects research (e.g. health research, medical research, cross-cultural psychology)**

Did you obtain written informed consent from a representative of the local community or region before the research took place? How did you establish who speaks for the community? Details of written informed consent obtained from study participants should be reported separately in the Methods section of your manuscript.

**Community Consent:**

Yes, written informed consent was obtained from a recognized representative of the local community prior to the commencement of the research. To determine who had the authority to speak on behalf of the community, we engaged with local government officials and community leaders, including the village executive officers and ward health officers. These individuals are formally appointed and widely acknowledged within the community as legitimate representatives and decision-makers.

Before data collection, we conducted preliminary meetings to explain the study's objectives, procedures, and potential impact. Upon reaching mutual understanding and agreement, a written informed consent form was signed by the designated community representative to affirm their approval and support for the research.

**Participant Consent:**

Details of the written informed consent obtained from individual study participants are reported separately in the Methods section of the manuscript, per ethical research practices. (Page 9)

How did members of the local community provide input on the aims of the research investigation, its methodology, and its anticipated outcome(s)?

**Community Engagement and Input:**

Local community input was obtained through meetings with village leaders, health workers, and community-based organization representatives. These sessions allowed us to present the research objectives, methods, and expected outcomes. Community feedback helped refine the methodology and ensured cultural appropriateness.

**Understanding of Consent Materials:**

Informed consent documents were translated into Swahili and explained in simple, clear language. Trained staff provided verbal explanations, especially for participants with low literacy. A back-translation process confirmed accuracy, ensuring materials were fully understood by all stakeholders.

When engaging with the local community, how did you ensure that the informed consent documents and other materials could be understood by local stakeholders?

How will the findings of the research be made available in an understandable format to stakeholders in the community where the study was conducted (e.g. via a presentation, summary report, copies of publications, etc.)? Please provide details of how this will be achieved.

**Ensuring Understanding of Consent Materials:**

To ensure local stakeholders understood the informed consent documents, all materials were translated into Swahili and written in simple, non-technical language. Trained research assistants explained the content verbally, allowing participants to ask questions. For participants with low literacy, oral consent procedures and thumbprints were used, ensuring clarity and informed participation.

**Dissemination of Findings to the Community:**

Yes, the findings will be shared with the community in an accessible format. This will include:

-A community presentation involving local leaders, health workers, and participants.

-A summary report in Swahili distributed to health facilities and community representatives.

-Copies of any published articles will be provided to local authorities and health institutions.

**Non-human subjects research using specimens/ animals collected as part of the study, or those housed in archival collections. Examples include archaeology, paleontology, botany and zoology.**

Did the permission you obtained from a local authority to perform the study include an agreement on access to outputs and benefit sharing? This may include procedures to enable fair distribution of the benefits and resources arising from the research performed. Please include any details of Prior Informed Consent and Benefit Sharing Agreements obtained. These may be required by field-specific regulations, for example the Convention on Biological Diversity (CBD) and the associated Nagoya Protocol.

N/A

If the material used in your study was imported, please A) provide the year it was imported and B) indicate whether permits were obtained to import/export the materials used, C) provide details of any permits obtained. If this information is not available, please indicate this.

N/A

If you used archival specimens, please state how the material used in your study was acquired by the institute it is held in and provide details of any permits obtained for the original excavations/ sample collection. If this information is not available, please indicate this.

N/A

How was the potential cultural significance of the materials collected in your study to local communities considered in your research design? Were Indigenous peoples and/or local researchers and institutions involved with archaeological excavations / collection of specimens? If so, please provide a description of their involvement.

**Consideration of Cultural Significance:**

The cultural relevance and sensitivity of the materials collected were carefully addressed during the research design phase. Local beliefs, practices, and norms, especially those related to health, childbirth, and traditional medicine were respected throughout the study. Guidance from community leaders, traditional birth attendants, and local health professionals was sought to ensure culturally appropriate framing of questions and respectful engagement with participants.

**Involvement of Local Researchers and Institutions:**

Yes, local researchers, healthcare providers, and academic institutions were actively involved in both the planning and implementation of the study. Their involvement ensured that the research process aligned with local customs, fostered community trust, and upheld ethical standards. Moreover, their contributions enriched the interpretation of findings and promoted meaningful, community-centred engagement.

If your manuscript includes photographs of human remains please indicate whether authors obtained permission from descendants or affiliated cultural communities to do so.

N/A
