# Supplementary material for: Prevalence and determinants of medicinal plants utilization during labour among women of reproductive age in Butiama, Tanzania: A community-based cross-sectional study
Source: PLoS One. 2025 Oct 31;20(10):e0334453. doi: 10.1371/journal.pone.0334453 (PMC12578257; doi:10.1371/journal.pone.0334453)
Supplement: Attachment — Submitted filename: S1 Rebuttal letter for Reviewers.docx [file pone.0334453.s005.docx]

**PONE-D-24-53690**

Factors influencing herbal medicine utilization during labour and delivery among women of reproductive age in Butiama, Tanzania: A community-based cross-sectional study

**Comments from the Editor**

| **S/N** | **Academic editor comments** | **Response** | **Page no:** |
| --- | --- | --- | --- |
| 1. | Please ensure that your manuscript meets PLOS ONE's style requirements, including those for file naming. The PLOS ONE style templates can be found at  https://journals.plos.org/plosone/s/file?id=wjVg/PLOSOne_formatting_sample_main_body.pdf and  https://journals.plos.org/plosone/s/file?id=ba62/PLOSOne_formatting_sample_title_authors_affiliations.pdf | Thank you for your reminder. We have reviewed the manuscript and ensured it aligns with PLOS ONE's style requirements, including the proper file naming conventions. We will ensure all future submissions adhere to these guidelines. | - |
| 2. | We note you have included a table to which you do not refer in the text of your manuscript. Please ensure that you refer to Table 4 in your text; if accepted, production will need this reference to link the reader to the Table. | Thank you for your observation. We have now included a reference to Table 4 in the relevant section of the manuscript to ensure proper linkage for the reader. | 14 |
| 3. | Please remove all personal information, ensure that the data shared are following participant consent, and re-upload a fully anonymized data set. | Thank you for your comment. We have carefully reviewed the dataset and ensured that all personal information has been removed in compliance with participant consent. The fully anonymized dataset has now been re-uploaded as requested. | - |

**Reviewer #1**

**Reviewer #1:** This is an interesting paper about the use of herbal medicine in Butiama, Tanzania during labour and delivery among women of reproductive age. The study is well done, I underline some brief points:

**Response:** Thank you for reviewing our paper, for your positive feedback, and for recognizing the significance of our study on the use of herbal medicine during labour and delivery among women of reproductive age in Butiama, Tanzania. We appreciate your thoughtful review and have addressed all the specific points and suggestions you highlighted, which have further strengthened our paper.

| **S/N** | **Comments from the reviewer #1** | **Response** | **Page no.** |
| --- | --- | --- | --- |
| 1. | The title does not reflect the entire content of the article; it needs to be reviewed. | Thank you for your valuable feedback regarding the title. We understand the importance of ensuring that it accurately reflects the full scope of the study. In response, we have revised the title to better capture the content and findings of the article: "Prevalence and Determinants of Herbal Medicine Utilization During Childbirth among Women of Reproductive Age in Butiama, Tanzania: A Community-Based Cross-Sectional Study." | 2 |
| 2. | Table 1 should be eliminated because the majority of the information has been repeated in Table 2. The latter is largely sufficient to present the results. | Thank you for your valuable feedback. As per your suggestion, we have eliminated Table 1, as the majority of the information is repeated in Table 2. We agree that Table 2 sufficiently presents the results, and we have made the necessary revisions accordingly. | 10 |
| 3. | Please correct in the Page 10, table 1 the value, ‘’never used HM = 165’’ et Utilization of HM during childbirth, No = 164)? | Thank you for pointing this out. We have corrected the values in Table 1 on page 10. The value for "never used HM" has been updated to 165. | 11 |
| 4. | Table 1: This question is not correct ‘’ Type of HM used during childbirth’’, because the proposed answers are varied. | Thank you for your feedback. We have addressed the concern regarding the question "Type of HM used during childbirth." In response, we revised the question to specifically inquire about the types of herbal medicine used (as we used during data collection). The revised options now include: Pumpkin roots (Cucurbita pepo), African spider plant (Cleome gynandra), Ginger (Zingiber officinale Roscoe), Concentrated green or black tea (Camellia sinensis), and "Never used herbal medicine."  **N:B Now it is shifted to Table 2** | 11 |
| 5. | In Table 2, the sum of the values for all the variables studied does not give 233, which is the total of participants who confirmed the use of plants. All the values in this table must be corrected. | Thank you for your valuable feedback. We have addressed the issue you raised regarding the sum of the values in Table 2 not adding up to 233, the total number of participants who confirmed the use of herbal medicine. The necessary corrections have been made to ensure that all values in the table are accurate and align with the total number of participants. Thank you once again for this valuable observation. | 10 |

**Reviewer #2**

**Reviewer #2:** Thank you for the possibility to read your interesting paper.

Assessing the statistics is challenging. I downloaded the Excel file and imported it to SPSS to check some data and calculations.

**Response:** Thank you for your feedback and for taking the time to review our paper. We have adjusted each relevant section and revised our analysis accordingly. We hope these revisions clarify any issues, and we are happy to provide further explanations if needed. We truly appreciate your thoughtful review and have addressed all the specific points and suggestions you highlighted, which have further strengthened our paper.

| **S/N** | **Comments from the reviewer #1** | **Response** | **Page no.** |
| --- | --- | --- | --- |
| 1. | First of all, I found that there seems to be several errors in Table 2. For “Age group” the percentages are mixed up (% for “all participants” come under “No”, % for “Yes” belong to “No” etc). This made me do the analysis in SPSS and I get different values for both counts and p-values for most of the factors in the table.  I struggle to see why you have chosen a significance level of 0.25. that is very high. And talking about significance, it is difficult to see what is significantly higher or lower than what. | Thank you for your insightful comment. We sincerely appreciate your thorough review of Table 2. Upon reviewing the table, we acknowledge that there were errors in the presentation of the percentages, where the values for "all participants," "No," and "Yes" were incorrectly assigned to the wrong categories. We have corrected these errors and recalculated the relevant counts and p-values to align with the correct data. Thank you again for bringing this to our attention, and we apologize for any confusion caused. The updated table now accurately reflects the findings. | 10  8 |
|  |  | Thank you for your valuable comment. The significance level of 0.25 was chosen for qualifying variables from the bivariate analysis to be entered into the multivariate analysis, as explained in the methodology section of the paper. This approach follows the guidelines established in reference No. 26 (Martinez et al., 2017), which suggests using a more liberal threshold for inclusion in multivariate models. |  |
|  | For Table 3 many confidence intervals are overlapping – does that mean anything for the results? | Thank you for your comment. We have reviewed the results and adjusted the analysis in the new version of the table to address the issue of overlapping confidence intervals. The updated version ensures a more accurate interpretation of the results. We appreciate your careful attention to this detail.  **N:B Now it is available as Table 4** | 13 |
|  | Table 4 has some numbers in bold and marked with stars – what do those markings refer to? They normally refer to significant values, but if that is the case here, what method is used for the analysis? Remember that a table should always be understandable in itself so this one needs some sub-text with explanations. | Thank you for your valuable feedback. To clarify, we employed descriptive analysis of univariate data to determine the distribution of responses based on the Likert scale. The bolded numbers in Table 4 highlight values of interest, and we have now explained these markings in the sub-text added to the table. This revision ensures that the table is fully self-explanatory. We appreciate your insights, and we believe these adjustments enhance the clarity of the table. Thank you once again for your constructive input! | 14 |
| 2. | Other comments:  A definition of “herbal medicine” would be useful. How did you explain to the participants what you were asking about? And is it necessary to make the abbreviation “HM”. | Thank you for your thoughtful comments. We appreciate your suggestion regarding the definition of “herbal medicine.” We have now included a clear definition in the manuscript to ensure that readers understand the term in the context of our study. Additionally, during data collection, we provided participants with a straightforward explanation of herbal medicine, describing it as ‘any plant-based substances, including leaves, roots, flowers, or teas, used to aid in labour and delivery without prescription from formal healthcare providers’.  Regarding the abbreviation “HM,” we recognize that it may not be essential. To enhance clarity and readability, we have decided to remove the abbreviation and instead use the full term “herbal medicine” throughout the manuscript. | Page 3  Page 7 |
| 3. | Where does reference 1 say that “conventional and alternative therapies contribute to 80% of global healthcare”? | Thank you for your comment. The information regarding the contribution of conventional and alternative therapies to global healthcare is available in the embedded file under the WHO reference. Additionally, we have included two other references that report similar findings. We have also refined the sentence to:  "The World Health Organization (WHO) and other studies report that nearly 50% of people in many industrialized countries use both conventional and alternative therapies, with usage rates reaching up to 80% in numerous developing countries." | 3 |
| 4. | You say that “Studies show that herbal medicine during labour induces intense, prolonged contractions….etc…” but which herbal medicines? It cannot be a general statement about herbal medicines. Reference 8 mentions one herb. | Thank you for your valuable feedback. We have addressed the concern regarding the specific herbal medicines by clarifying which herbs are associated with the complications mentioned and referencing the relevant studies that detail their effects. The revised text now includes plants used to induce labour, such as oxytocic herbs like the African spider plant (Cleome gynandra), Cissampelos mucronata, Mwanamphepo (Cissus species), Ampelocissus, Ginger and Cyphostemma. We appreciate your suggestions and believe these revisions enhance the clarity and accuracy of our paper. | 3 |
| 5. | What does the number of modes of administration have to do with the cultural significance of herbal medicine? | Thank you for your comment. We have removed the reference to the number of modes of administration as it was not directly relevant to the cultural significance of herbal medicine. We appreciate your feedback and believe this revision improves the clarity of the paper. | 3 |
| 6. | Your section about training research assistants is rather detailed. | Thank you for your feedback. We have addressed the concern by streamlining the section on training research assistants. The revised version now presents the key points more concisely while retaining all necessary information about the training process. | 7 |
| 7. | Ethics: I guess you mean “Muhimbili University of Health and Allied Sciences”. | Yes, you are correct. We meant "Muhimbili University of Health and Allied Sciences." Thank you for pointing that out. | 8 |
| 8. | **Results:**  Response rate: This is not about how many you aimed to include but about how many you asked to participate versus how many were willing. | Thank you for your comment. We have revised the statement to accurately reflect the response rate. It now reads: "A total of 406 participants were initially approached for the study, with 398 providing informed consent, yielding a response rate of 98.03%."  Thank you again for your feedback. | 9 |
| 9. | Please reconsider how you write the sentences with numbers and percentages – it does not flow as it is now. | Thank you for your feedback. We have revised the section to ensure the presentation of numbers and percentages flows more smoothly. | 9,10,11 &12. |
| 10. | I suppose you KNOW how many participants used herbal medicines during childbirth – not “around 233” (which is an approximate number) but “233”. The same goes for “About 272” participants with primary education – it is 272 – not “about”. | Thank you for your observation. You are correct, and we appreciate the clarification. The numbers should indeed be exact rather than approximate. We have revised the text accordingly i.e. A total of 233 (58.5%) participants used herbal medicine during childbirth. | 9 |
| 11. | Why do you mention “roots” and “leaves” but not which roots or leaves? Does this matter? Is it interesting? Why? | Thank you for your comments. We have addressed the concern regarding the question "Type of HM used during childbirth." In response, we revised the question to specifically inquire about the types of herbal medicine used (as we used during data collection). The revised options now include: Pumpkin roots (Cucurbita pepo), African spider plant (Cleome gynandra), Ginger (Zingiber officinale Roscoe), Concentrated green or black tea (Camellia sinensis), and "Never used herbal medicine." | 11 |
|  | Discussion:  The statement “Women’s desire to maintain marriages,….. Reliance on HM” requires a reference. | Thank you for your comment. We have removed the mentioned statement as it is no longer applicable following the adjustment of our revised analysis. This change ensures that the content aligns with the updated findings and maintains clarity in the discussion. | 15 |
|  | Significance of educational level: there is a significant difference between those with none (reference) and those with primary education, but what can you say about those with primary vs. those with secondary education? | Thank you for your insightful comment. In the refined version of our analysis, the difference between those with primary education and those with secondary education is no longer statistically significant. This change was made after reassessing the data, and we have adjusted the results accordingly. We appreciate your attention to this detail and have made the necessary revisions to reflect the updated findings. | 16 |
|  | Overlapping confidence intervals – does that matter? Confidence intervals including “1” – does that matter? | Thank you for your question. Overlapping confidence intervals and confidence intervals that include "1" can indeed have implications for the interpretation of the results.  We have reviewed and addressed these points in the updated version of the manuscript to ensure the interpretation is clear and accurate. | 14 |
|  | Please also revise the abstract accordingly. | Thank you for your suggestion. We have revised the abstract accordingly to reflect the changes made in the whole manuscript. Thank you again for your helpful feedback. | 2 |

Best regards,

Corresponding author
