# Supplementary material for: Prevalence and determinants of medicinal plants utilization during labour among women of reproductive age in Butiama, Tanzania: A community-based cross-sectional study
Source: PLoS One. 2025 Oct 31;20(10):e0334453. doi: 10.1371/journal.pone.0334453 (PMC12578257; doi:10.1371/journal.pone.0334453)
Supplement: Attachment — Submitted filename: S1 rebuttal letter to Reviewers.docx [file pone.0334453.s006.docx]

**PONE-D-24-53690**

Factors influencing herbal medicine utilization during labour and delivery among women of reproductive age in Butiama, Tanzania: A community-based cross-sectional study

**Comments from the reviewers**

**Reviewer #2**

| **S/N** | **Comments reviewer #2** | **Response** | **Page no.** |
| --- | --- | --- | --- |
| 1 | Please remember that the number of decimals indicates the accuracy of the number given. You seem to be using one, two or three decimals randomly. Results: Lower education p=0,094 is said to be significant – is it? | Thank you for your comment. We hope this observation was based on a previous version of the manuscript. In the current version, education was not statistically significant in influencing the use of medicinal plants. Significant associations were observed with marital status, occupation, distance to the nearest health facility, number of antenatal care visits, and parity. All p-values and related statistics have been consistently reported using three decimal places throughout the revised manuscript. | - |
| 2 | How do you know that the “adverse effects” mentioned are actually adverse effects of the herbs used? They could be “suspected adverse effects” but do you know for sure that they ARE? | Thank you for this important observation. You are correct — we cannot definitively attribute the reported effects to the herbs used, as we did not conduct clinical assessments to confirm causality. Therefore, in the revised manuscript, we have clarified that these are self-reported suspected adverse effects associated with herbal use during pregnancy, as perceived by the participants. This distinction has been emphasized to avoid over interpretation and to acknowledge the limitations of self-reported data in the absence of clinical verification. | 11 |
| 3 | Please consider and discuss confounders: Is “living more than 5 km from nearest clinic” and “attending fewer than 4 ANC visits” the same thing? Or at least interconnected? | Thank you for this insightful comment. We agree that living more than 5 km from a health facility and attending fewer than four ANC visits may be interconnected and potentially act as confounding factors. Limited proximity to healthcare services can reduce access and frequency of ANC attendance, thereby influencing both variables simultaneously. In our analysis, these were treated as independent predictors; however, we acknowledge that geographic access may partially explain lower ANC attendance, which in turn could increase reliance on herbal remedies. We have added this consideration in the discussion to reflect the possible interrelationship and its implications for the interpretation of findings. | 16 |
| 4 | Table 3 needs a comment in the subtext explaining the meaning of the And in the top line you have to put COR [95% CI] and AOR [95% CI]. | Thank you for your observation. We have revised Table 3 to include a clear header indicating COR [95% CI] and AOR [95% CI]. Additionally, we have added a subtext note below the table explaining that COR refers to Crude Odds Ratio, AOR to Adjusted Odds Ratio, and CI to Confidence Interval. AOR accounts for other variables in the model. We hope this clarification improves the table’s interpretability. | 13 |
| 5 | - Community education on herbal remedy risks………………..complications.  - Additionally, involving community leaders, ……………..maternal care.  - Enhancing community education on the safe use…….informed choices.  I agree that the “community based” approach is an advantage. It is not very well known in Europe so a comment on WHY it is a strength would be good. | Thank you for the insightful comment. We agree that the community-based approach is an advantage, especially in low-resource settings like rural Tanzania. We have clearly stated its importance in the manuscript—it allows direct engagement with women in their own environments, helps capture culturally rooted practices such as herbal medicine use, and reaches populations often missed by facility-based studies. This context-specific approach enhances the relevance and applicability of the findings to local maternal health interventions. | 18 |
| **REVIEWER #3** | | | |
| **S/N** | **Comments reviewer #3** | **Response** | **Page no.** |
| 1 | Reviewer #3: Dear corresponding author The topic is relevant and interesting; however, the authors should address the following points to improve the manuscript: | We sincerely thank you for the positive feedback and for recognizing the relevance and importance of our study. We appreciate your constructive comments and have carefully addressed each of the points you raised to improve the quality of our manuscript. Detailed responses to your specific comments are provided below | - |
| 2 | The statement of the study’s purpose at the end of the introduction (last three lines, reference 23) is confusing and appears redundant. This phrasing is more appropriate for a research proposal rather than a journal article introduction. I recommend deleting this sentence and instead refining the purpose as stated earlier in the introduction. | Thank you for this insightful comment. We agree that the original phrasing of the study’s purpose at the end of the introduction was more suited to a research proposal format and may have introduced redundancy. In response, we have deleted the final sentence containing reference 23 and have refined the earlier purpose statement in the introduction to ensure clarity and alignment with journal article conventions. | 4 |
| 3. | In the Materials and Methods section, given that this is a cross-sectional (descriptive) study, the use of terms such as "independent variable" and "dependent variable" is inappropriate. Cause-and-effect relationships are not applicable in descriptive studies but rather in cohort, case-control, or intervention designs. It would be better to refer to all variables as contextual or descriptive. | Thank you for this valuable observation. We fully agree with your point that the terminology used should align with the study design. In response, we have revised the Materials and Methods section by removing references to “independent” and “dependent” variables. Instead, we now refer to the variables as “contextual” and “descriptive,” which is more appropriate for a cross-sectional descriptive study. | 7 |
| 4 | The description of obtaining written informed consent from unmarried participants under 18 years old is unclear. Since the inclusion criterion requires prior childbirth experience, it is questionable whether unmarried individuals under 18 would meet this criterion, especially considering cultural contexts such as those in many Middle Eastern countries. The authors should clarify this point in the manuscript. | Thank you for this important comment. We acknowledge the need for clarification. In our study setting (Tanzania), early childbirth typically occurs within the context of marriage or informal unions. Accordingly, all participants under 18 who met the inclusion criteria had prior childbirth experience and were either married or in such unions. We have revised the manuscript to reflect this and clarify the consent procedures used for minors. | 8 |
| 5 | The study population consists of women who gave birth between 2021 and 2024, with data collected from June to July 2024. Given this timeline, recall bias is a potential concern, particularly regarding herbal medicine use during childbirth. The authors should discuss how recall bias was addressed or minimized and provide more information on the validity of the questions related to herbal medicine use. | Thank you for this insightful comment. We would like to clarify that the study population included women who gave birth between 2023 and 2024, not from 2021 to 2024 as previously stated. This shorter recall period helped reduce the potential for recall bias. | 4 & 6 |
| 6 | The distinction between herbal medicines and medicinal plants needs clarification. Herbal medicines typically refer to products that have undergone formulation processes (e.g., medicines, syrups, creams), whereas medicinal plants are raw or minimally processed. The authors should specify their definition and approach regarding this distinction. | Thank you for this important comment. In our study, the term "herbal medicine" was used broadly to refer to plant-based substances consumed in raw, boiled, or infused form, without professional medical prescription or standardized formulation. These included locally recognized medicinal plants such as pumpkin roots, African spider plant, ginger, and concentrated tea, used traditionally during labour and delivery.  To avoid confusion, we have revised the manuscript to clarify that our focus was on the use of raw or minimally processed medicinal plants, aligning more closely with the reviewer’s definition. This clarification has been added to the operational definitions in the Methods section. | Entire manuscript  6 |
| 7 | The limitations of the study should be presented before the conclusions. Furthermore, the conclusions and recommendations should be aligned strictly with the study’s actual findings.  Overall, the article is suitable for publication after major revisions addressing the above points.  Thank you for considering these comments.  Sincerely, | Thank you for your constructive summary and valuable suggestions. In response, we have revised the structure of the manuscript by presenting the limitations section before the conclusions, as recommended. We have also carefully reviewed and revised the conclusions and recommendations to ensure they are directly aligned with the study’s findings without overgeneralization. We appreciate your thoughtful feedback, which has helped improve the quality and clarity of the manuscript. | 18 & 19 |
