# Supplementary material for: Prevalence and determinants of medicinal plants utilization during labour among women of reproductive age in Butiama, Tanzania: A community-based cross-sectional study
Source: PLoS One. 2025 Oct 31;20(10):e0334453. doi: 10.1371/journal.pone.0334453 (PMC12578257; doi:10.1371/journal.pone.0334453)
Supplement: Attachment — Submitted filename: S1 Rebuttal letter to Reviewers..docx [file pone.0334453.s007.docx]

**PONE-D-24-53690R2**

Prevalence and determinants of medicinal plants utilization during labour among women of reproductive age in Butiama, Tanzania: A community-based cross-sectional study

**General response to both reviewers:** We sincerely thank the reviewers for their valuable and constructive feedback, which greatly improved the quality and clarity of our manuscript. All suggestions and concerns have been carefully addressed and incorporated into the revised version. We truly appreciate your time and thoughtful review.

**Reviewer #2**

| **SN** | **Reviewer’s comment** | **Response** | **Page.** |
| --- | --- | --- | --- |
| 1. | Please check once again for spelling mistakes. Please be consistent: write all Latin names in italics (f.x. page 3 and Table 2, but could be anywhere), English/African names should NOT be in italics (f.x. page / "Main outcome variables", but could be other places as well). | Thank you for your observation. We have carefully reviewed the manuscript and ensured that all Latin (scientific) names are consistently italicized throughout, including in Table 2 and on page 3. In contrast, all English and African names remain in regular (non-italicized) text. We have also thoroughly checked the manuscript for spelling errors and corrected them accordingly. | 3, 7, 12. |
| 2. | "...pregnancy and or labour" should be "pregnancy and/or labour". | Thank you for pointing that out. We have corrected the phrasing from "pregnancy and or labour" to "pregnancy and/or labour" throughout the manuscript to ensure clarity and proper punctuation. | The whole manuscript |
| 3. | All tables and figures should be understandable "in themselves".  Table 2: "suspected adverse effects" - not only in text but in the table too. | Thank you for the helpful suggestion. We have revised all tables to ensure they are fully understandable on their own, without needing to refer to the main text. Specifically, in Table 2, we have clarified the term “suspected adverse effects” by explicitly including it within the table content | 12 |
| 4. | Page 12 "Predictors of ....": numbers of decimals says something about how exact a number is - please be consistent and perferably use no more than one decimal when you say that "X had x.y times higher odds than Y" (to be "1.823 times more likely to use medicinal plants" is overdoing it - 1.8 would be fine). And please change in the abstract too. | Thank you for the insightful comment. We have revised the decimal formatting throughout the manuscript, including on page 12 and in the abstract, to ensure consistency and appropriate precision. Specifically, we now report odds ratios and similar values with no more than one decimal place | 2 and 13 |
| 5. | Table 3: what does the * refer to? Needs to be explained below the table. And I still think it is incorrect to say that "distance" and "number of visits" are both significant as they are most probably interrelated. A comment would be good. | Thank you for your observation. We have added a footnote below **Table 3** to explain that the asterisk (*) indicates statistical significance at **p < 0.05**.  Additionally, we acknowledge the potential interrelation between **distance to the nearest health facility** and the **number of ANC visits**. While both variables showed statistical significance in the multivariable model, we have added a comment in the discussion section to note the possibility of collinearity and to interpret the findings with caution, as reduced access due to distance may influence ANC attendance. | 14  17 |
| 6. | Page 14: "Perceptions and beliefs...": I don't see 48.7 in the table? Maybe say that"... 48.7% strongly agreed or agreed...". The same goes for the other numbers. | Thank you for your observation. We have revised the text on page 14 to clearly state that the percentages refer to participants who **strongly agreed or agreed** with each statement, ensuring consistency with the data presented in Table 4. This clarification has been applied throughout the relevant paragraph to improve accuracy and interpretation. | 15 |
| 7. | Table 4: The sub-text about "bolded values" is unclear - please rephrase. Items 2 and 3:" ... medicinal plants are ...." | Thank you for your helpful comments. We have revised the sub-text in Table 4 to clarify the meaning of the bolded values. It now clearly states that **Bolded values indicate the most frequent responses, reflecting participants’ dominant views.** Additionally, we have corrected the phrasing in items 2 and 3 to read: *“...medicinal plants are...”* for grammatical accuracy and consistency. | 15 |

| **Reviewer #3** | | | |
| --- | --- | --- | --- |
| 1. | Given that participants were asked about their use of medicinal plants "during labour and delivery," I would like to clarify how this period was defined for the participants to avoid confusion with the pregnancy period. Could you please provide an operational definition of "labour and delivery" as used in your study? Specifically, from what point in time was this period considered to begin and end. | Thank you for your comment. In our study, **“labour and delivery”** was operationally defined as the period starting from the onset of regular, painful uterine contractions signalling the beginning of active labour, continuing through the delivery of the baby and the placenta. This period was clearly distinguished from the pregnancy period to ensure participants understood that any medicinal plant use reported specifically referred to the time during active labour and/or childbirth, not earlier stages of pregnancy. | 7  8, 9 |
|  | The use of medicinal plants for labor induction, labor acceleration, or other purposes during pregnancy among women with previous pregnancy and childbirth experience is still unclear. Given that participants were asked about the purpose of use, we kindly request that you define and clarify the operational meaning of "labor induction and acceleration" in the Materials and Methods section of the manuscript. | Thank you for your valuable feedback. We appreciate your suggestion and have addressed it accordingly. In the Materials and Methods section, we have now included the following operational definitions:  In this study, labour induction refers to the use of medicinal plants with the intent to initiate uterine contractions before the spontaneous onset of labour. Labour acceleration refers to the use of medicinal plants to hasten the progress of labour once it has already begun, with the goal of shortening the duration of childbirth. These definitions were clearly explained to participants during data collection to ensure consistent understanding. |  |
| 2. | The sample size should be clearly specified in Table 3. | Thank you for your comment. We have now clearly specified the sample size (n=398) in the title/header of Table 3 for clarity. All tables have been revisited | 14 |
| 3. | What are the study limitations? Please address potential issues such as participants possibly misunderstanding the questions, external factors like time constraints, resource availability, or local conditions, variations in sampling or data collection methods, and even the sample size itself. | Thank you for your insightful comments. We have expanded the limitations section to address potential issues such as participant misunderstanding despite careful questionnaire design and pilot testing. External factors like time constraints, limited resources, and local conditions may have influenced data quality. Although standardized protocols were used, minor variations in sampling and data collection across sites are possible. Additionally, the cross-sectional design limits causal inference, and the sample size may affect the generalizability of our findings. These points are now clearly acknowledged in the revised manuscript. | 19 |
| 4. | The data collection method needs to be described more clearly and with greater detail.  Considering the participants’ educational levels and literacy, was a uniform data collection method applied to all? Please provide further explanation | Thank you for this important comment. We would like to emphasize that a uniform, interviewer-administered questionnaire was used for all participants to ensure consistency, regardless of their educational level or literacy. All interviews were conducted face-to-face in the Kiswahili language by trained research assistants, who used simple language and culturally appropriate examples to enhance understanding, particularly for key concepts like “medicinal plants.” This approach helped ensure accurate and inclusive responses. | 8 |
| 5. | Regarding the reviewer’s previous comment: "A definition of 'herbal medicine' would be useful. How did you explain what you were asking about to the participants? And is it necessary to abbreviate it as 'HM'?" — | Thank you for your valuable suggestion. Since the last revision, we have replaced the abbreviation "HM" with the full term **"medicinal plants"** throughout the manuscript, as recommended. We have also added a clear operational definition in the Methods section to guide participant understanding. Specifically, participants were informed that *medicinal plants* referred to any plant-based substances consumed in raw or minimally processed form without a prescription or recommendation from a healthcare provider, and used during labour and/or delivery. Common examples cited included pumpkin roots (*Cucurbita pepo*), African spider plant (*Cleome gynandra*), ginger (*Zingiber officinale Roscoe*), and concentrated green or black tea (*Camellia sinensis*). This explanation was provided during data collection to ensure clarity and consistency in participants' responses (available in Methods and Materials section, Outcome Variables). | 7 |
| 6 | The authors’ response, the explanation provided seems more useful for the readers of the article rather than addressing how the concept was explained to the participants. Although the authors gave good explanations to help participants understand, these may still be insufficient, as participants might have varied interpretations. If this is the case, it should be acknowledged as a limitation of the study. | Thank you for this valuable observation. While the definition of medicinal plants was provided, we agree it may not fully reflect participants' interpretations. During data collection, interviewers used simple, locally relevant examples and clarified that medicinal plants referred to any raw or minimally processed plant substances used during labour without medical advice. Despite these efforts, interpretations may have varied. We have therefore revised the **Limitations** section of the manuscript to explicitly acknowledge this potential issue as a source of response variability. | 19 |
| 7. | Regarding the validity of the questionnaire on participants’ perceptions and beliefs about the use of medicinal plants during childbirth, which was evaluated by five experts in public health, maternal and child health, and linguistics, the reported content validity index (CVI) of 0.90 for each item and over 0.90 overall (0.93) requires further clarification. Specifically, how was the 3% increase in the overall CVI achieved?  Additionally, considering the internal consistency of the questions, with a Cronbach’s alpha coefficient of 0.84, it is expected that the results section—especially the part related to Table 4—will include a classification of participants’ beliefs into levels such as weak, average, and good, or any other categorization the authors find appropriate. This classification would enhance the discussion and interpretation of the findings. | Thank you for your comment. The overall CVI of 0.93 was achieved because several items received perfect relevance scores (I-CVI = 1.00) from all five experts, which slightly raised the average above 0.90 due to rounding up. The Cronbach’s alpha of 0.84 confirmed good internal consistency. Based on this, we categorized participants’ perceptions in Table 4 using Likert scale levels (e.g., strongly disagree to strongly agree) to enhance interpretation, as recommended. | 7, 8 |

Best regards,

Corresponding author.
