# Supplementary material for: Prevalence and determinants of medicinal plants utilization during labour among women of reproductive age in Butiama, Tanzania: A community-based cross-sectional study
Source: PLoS One. 2025 Oct 31;20(10):e0334453. doi: 10.1371/journal.pone.0334453 (PMC12578257; doi:10.1371/journal.pone.0334453)
Supplement: Attachment — Submitted filename: S1 Response to reviwers.docx [file pone.0334453.s008.docx]

**PONE-D-24-53690R2**

Prevalence and determinants of medicinal plants utilization during labour among women of reproductive age in Butiama, Tanzania: A community-based cross-sectional study

| **SN** | **Reviewer’s comment** | **Response** | **Page.** |
| --- | --- | --- | --- |
|  | Title and Abstract  Statement: In general, the title and abstract were sufficiently informative, with the caveat that the abstract could be tightened up.  Explanation: The authors repeated "labour and/or delivery" to a fault, which overall resulted in a wordy paper, in part because the ambiguous end to their text.  Example: The summary sentence for this section could have simply been: "Over fifty percent of participants reported utilizing medicinal plants during childbirth, with various socio-economic status and health care access factors indicating that education and interventions targeted around medicinal plants would be advantageous. | We appreciate the reviewer’s observation regarding the repeated use of “labour and/or delivery,” which contributed to wordiness. In response, we have revised the summary sentence for clarity and conciseness as suggested. | 2 |
|  | Introduction  Statement: Overall, I thought the introduction was decent, but a bit bogged-down with bloated content.  Explanation: The sheer number of plants (some with awkward language (helpful info overall), broke my flow and allowed me to disengage with it.  Example: I think you can talk about prevalence and risk factors more than you can plant descriptions. You could have situated plant examples in the Discussion. | Thank you for your feedback. The plant descriptions were included in the Introduction to provide essential contextual background. After discussion with co-authors and other experts, it was agreed that this information is necessary early to help readers understand the study’s context, alongside prevalence and risk factors. | 3 |
|  | Methods  Statement: Overall, I thought the reporting approach was strong, and my understanding of the reporting was clear, but there were areas that were unnecessary details that went above and beyond relevance to the study.  Explanation: The ethical considerations and validation testing were aptly described, but it seems the authors devoted a substantial amount of detail to more lengthy operational definitions of "labour induction/acceleration".  Example: The broadness of "labour induction/acceleration", or detailed specificities could have been included in the results section with the other operational definitions in a paragraph of precise text. | Thank you for your feedback. The detailed operational definitions of "labour induction/acceleration" were included in the Methods to ensure clarity and reproducibility of the study procedures. We recognize that some details could alternatively be summarized in the Results, but we felt it was important to define them upfront for transparency and to avoid ambiguity in interpretation. |  |
|  | **Results**  Statement: Overall the results were clear, with identified predictors and estimates of prevalence, but could have been expressed more efficiently.  Explanation: Mostly the results section was legibility by primarily expressing some details in both the tables as well as in the text.  Example: Highlight the key predictors and odds ratios as indicated for example "Women who used less than 4 ANC visits (AOR=0.556, p=0.006)" were more likely to use medicinal plants. The participants were asked every percentage? | The use of percentages alongside odds ratios is justified because percentages describe the prevalence of an outcome in the study population, while odds ratios quantify the strength of association between predictors and the outcome. Including both allows readers to understand not only how common a behavior or characteristic is but also which factors significantly influence it. Percentages provide context for interpreting the magnitude of the odds ratios and make the results more interpretable and meaningful. | 14 |
|  | **Discussion**  Statement: The discussion is thorough and fairly contextualized, perhaps too extensive.  Explanation: Comparisons from region to region were useful for comparisons, however sometimes they pulled away the uniqueness of this study.  Example: It would help to remain focused on the three themes, explain socio-economic / cultural factors, and access to health care and ANC utilizations, and be explicit regarding the unique findings for Butiama district. | We appreciate the reviewer’s feedback. After consideration, we have decided to retain the discussion as presented, as the regional comparisons provide useful context. We have ensured the discussion remains focused on the three main themes, addresses socio-economic, cultural, and health care access factors, and highlights participants’ perceptions of medicinal plant use. | 15-19 |
|  | Limitations  Statement: The authors are honest limitations, although overall fairly lengthy.  Explanation: Some details (environmental conditions) fails to be relevant overall your manuscript.  Example: Can summarize: The main limitations were cross-sectional and recall bias, and the potential misinterpretation of 'medicinal plants'even with clear explanation from interviewer. | We thank the reviewer for the valuable comment. We agree that some of the details on environmental conditions may not be directly relevant to the overall manuscript. We have revised the limitations section to focus on the key points: the cross-sectional design, potential recall bias, and the possible misinterpretation of “medicinal plants” despite explanations provided by the interviewer. | 19 |
|  | Conclusions and Recommendations  Statement: The conclusion was solid, with some degree of verbal blurring with the recommendations.  Explanation: It would be useful to gain a little more distance from our findings to make suggestions, but the recommendations would still be sectioned distinctly from the conclusions.  Example: Conclude the statements summarized in the conclusion with, "This study brought timely attention to the importance of speaking about the use of medicinal plants as we provide maternal health services." and just keep the recommendations as a section. | We thank the reviewer for the constructive comment. We agree that the conclusions and recommendations should be more clearly separated. We have revised the conclusion to summarize the main findings and added the suggested statement: *“This study brought timely attention to the importance of speaking about the use of medicinal plants as we provide maternal health services.”* The recommendations have been retained as a distinct section to ensure clarity and focus. | 20 |
|  | References  Statement: Overall the references are appropriate to the context of the research and largely current, however some were lesser sources.  Explanation: In general, less of the websites or unlabelled attribution would be helpful in not pulling from potential sources.  Example: Source potential from other peer-reviewed literature (i.e., the systematic reviews that review herbal medicine use during childbirth) or methodologies. | We thank the reviewer for this observation and agree with the comment regarding the references. While most references included in the study are appropriate and current, we acknowledge that some sources, particularly certain websites or those with unclear attribution, may be less reliable. We have done a review to prioritize peer-reviewed literature, including systematic reviews on herbal medicine use during childbirth and relevant methodological references, to strengthen the credibility and scholarly rigor of the study. | 22-25 |
|  | Overall, the manuscript is methodologically rigorous, ethically sound and a contribution to the knowledge in maternal health (and maternal health research) literature base in sub-Saharan Africa. | Thank you for your positive feedback. I’m glad the study is considered a valuable contribution to maternal health research in sub-Saharan Africa. | - |
|  | **Reviewer #2:** |  |  |
| 1. | Dear Authors, Now I only have a very few comments on the manuscript, as you have done a good job revising it.  Please check for a few more "and or" which should be "an/or". | Thank you for your positive feedback on the revised manuscript. We have carefully checked the text again and corrected the remaining instances of "and or" to "and/or" as suggested. | Whole manuscript |
| 2. | First line in Introduction: The World Health Organisation (WHO) and various (not "other" as WHO is not a study). | Thank you for this helpful observation. We have revised the first line in the Introduction to read: *“The World Health Organisation (WHO) and various studies …”* instead of *“other studies”* to ensure accuracy. | 3 |
| 3. | Same page, line 11: ".... and increased risk of cesarean section" (or increased number of cesarean...) | Thank you for pointing this out. We have revised the sentence on page 3, line 11 to improve clarity. It now reads: *“… and an increased risk of cesarean section.”* | 3 |
| 4. | In Materials and methods, Sampling technique, First stage you talk about "wards" - is that the right word? Or is it "clinics"? | Thank you for the comment. The correct unit of selection was administrative wards, not clinics. Wards were chosen because they are the official administrative subdivisions of Butiama Council and served as the primary sampling units. We have clarified this in the Materials and Methods section. | 6 |
| 5. | Main outcome variables, you mention "pumpkin roots" - are the roots actually used? Or the pumpkin it self or the seeds? | Thank you for your comment. Yes, in this locality, **pumpkin roots** are the part traditionally used and are reported to have labor-expediting effects. The fruit and seeds are not used for medicinal purposes. | 7 |
| 6. | Pre-testing of data collection instruments: the questionnaire was administered "orally" - not "verbally". | Thank you. We have replaced 'verbally' with 'orally' to accurately indicate that the questionnaire was read aloud to participants. | 8 |
| 7. | Table 2: Here it still says "to enhance labour" (should be "hasten" as in the text above the table). | Thank you. We have corrected Table 2 to replace 'to enhance labour' with 'to hasten labour' to ensure consistency with the text. | 12 |
| 9. | With those minor changes, I think your paper is ready for publishing. | Thank you very much for your review and guidance. We appreciate your feedback and are glad that the paper is now ready for publishing. | - |
|  |  |  |  |
|  | **Reviewer #3: Dear Author**  Please ensure all abbreviations are spelled out in full when first used in the abstract, main text, or footnotes for clarity. | Thank you for your comment. We have ensured that all abbreviations are spelled out in full when first used in the abstract and main text | Entire manuscript |
